# Optimal Isolation of Functional Foxp3^+^ Induced Regulatory T Cells Using DEREG Mice

**DOI:** 10.1371/journal.pone.0044760

**Published:** 2012-09-05

**Authors:** Abdul Mannan Baru, Christopher Untucht, Venkateswaran Ganesh, Christina Hesse, Christian T. Mayer, Tim Sparwasser

**Affiliations:** Institute of Infection Immunology, TWINCORE, Centre for Experimental and Clinical Infection Research; A Joint Venture between the Medical School Hannover (MHH) and The Helmholtz Centre for Infection Research (HZI), Hannover, Germany; Friedrich-Alexander-University Erlangen, Germany

## Abstract

Foxp3 reporter mice including DEREG (DEpletion of REGulatory T cells) mice have greatly helped in exploring the biology of Foxp3^+^ Tregs. DEREG mice express a DTR-eGFP fusion protein under the control of a bacterial artificial chromosome (BAC)-encoded Foxp3 promoter, allowing the viable isolation and inducible depletion of Foxp3^+^ Tregs. Adaptive Tregs differentiated *in vitro* to express Foxp3 (iTregs) are gaining high interest as potential therapeutics for inflammatory conditions such as autoimmunity, allergy and transplant rejection. However, selective isolation of Foxp3^+^ iTregs with a stable phenotype still remains to be a problem, especially in the human setting. While screening for culture conditions to generate stable CD4^+^Foxp3^+^ iTregs from DEREG mice, with maximum suppressive activity, we observed an unexpected dichotomy of eGFP and Foxp3 expression which is not seen in *ex vivo* isolated cells from DEREG mice. Further characterization of eGFP^+^Foxp3^−^ cells revealed relatively lower CD25 expression and a lack of suppressive activity *in vitro*. Similarly, eGFP^−^ cells isolated from the same cultures were not suppressive despite of a broad CD25 expression reflecting mere T cell activation. In contrast, eGFP^+^Foxp3^+^ iTregs exhibited potent suppressive activity comparable to that of natural eGFP^+^Foxp3^+^ Tregs, emphasizing the importance of isolating Foxp3 expressing iTregs. Interestingly, the use of plate-bound anti-CD3 and anti-CD28 or Flt3L-driven BMDC resulted in considerable resolution of the observed dichotomy. In summary, we defined culture conditions for efficient generation of eGFP^+^Foxp3^+^ iTregs by use of DEREG mice. Isolation of functional Foxp3^+^ iTregs using DEREG mice can also be achieved under sub-optimal conditions based on the magnitude of surface CD25 expression, in synergy with transgene encoded eGFP. Besides, the reported phenomenon may be of general interest for exploring Foxp3 gene regulation, given that Foxp3 and eGFP expression are driven from distinct Foxp3 loci and because this dichotomy preferentially occurs only under defined *in vitro* conditions.

## Introduction

Foxp3 is an established marker for the identification of both natural and induced CD4^+^ regulatory T cells (Tregs) [1]–[4], yet it is inaccessible to reagents for their viable isolation or depletion. To overcome this limitation, DEREG mouse was generated which report Foxp3 promoter activity by the expression of a DTR-eGFP fusion protein from an ectopic bacterial artificial chromosome (BAC)-encoded Foxp3 locus [5]. The use of Foxp3 reporter mice, including DEREG mice have firmly established the crucial and non-redundant role of CD4^+^Foxp3^+^ Tregs in preserving the immune homeostasis and maintaining immunological self/tumor-specific tolerance [6]–[11]. Consequently, CD4^+^Foxp3^+^ Tregs are gaining impetus as prophylactics or therapeutics in order to regulate various immune disorders such as transplant rejection, autoimmunity and allergy. Nevertheless, in many instances the number of Tregs required for an effective intervention proves to be a limitation for their application. Recent advances pertaining to *ex vivo* induction and expansion of Foxp3^+^ Tregs (iTregs) from naïve CD4^+^Foxp3^−^ T cells in the presence of TGF-β and retinoic acid (RA) [12],[13] could potentially surmount this bottleneck. Isolation and transfer of Tregs on the basis of classical Treg surface markers (e.g. CD25), which simultaneously get strongly up-regulated on conventional T cells during *in vitro* activation, poses a potential risk for their clinical application. Additionally, employment of polyclonal or antigen-specific Foxp3^+^ iTregs as potential therapeutics is a matter of debate [14],[15]. Besides, Foxp3^+^ iTregs generated *in vitro* tend to rapidly lose Foxp3 expression and concomitantly their suppressive activity following adoptive transfer [16],[17]. Consequently, culture conditions favoring the induction of stable Foxp3 expression as well as strategies for the selective isolation of Foxp3^+^ iTregs from these cultures remain to be established. In this study, we report protocols for the optimal generation and isolation of functional eGFP^+^Foxp3^+^ iTregs using DEREG mice.

## Results

**Figure 1:**
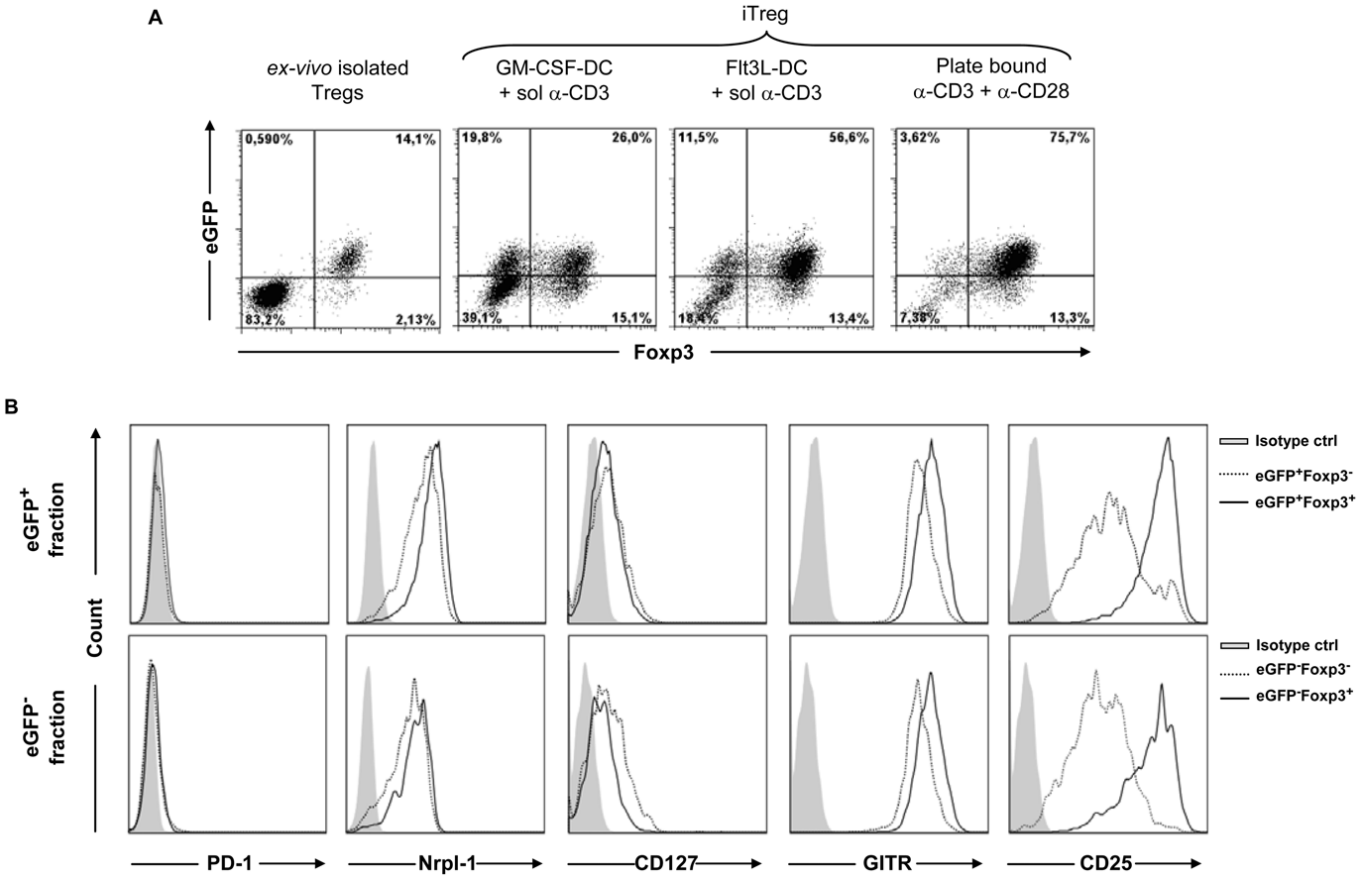
Culture conditions demonstrating eGFP and Foxp3 expression dichotomy amongst in vitro generated iTregs from DEREG mice. (A) Dot plots display eGFP and Foxp3 expression among the live CD4^+^ gated DEREG cells. Shown are *ex vivo* stained T cells (left), *in vitro* differentiated iTregs using soluble anti-CD3, TGF-β, RA and GM-CS- or Flt3L-derived BMDC (second and third panels respectively) and iTregs using plate-bound anti-CD3, anti-CD28, TGF-β and RA (right). (B) Differential expression of various surface antigens on eGFP^+^Foxp3^−^ (dotted line, upper panel) and eGFP^+^Foxp3^+^ (solid line, upper panel) iTregs and eGFP^−^Foxp3^−^ (dotted line, lower panel) and eGFP^−^Foxp3^+^ (solid line, lower panel) cells differentiated in the presence of TGF-β, RA, soluble anti-CD3 and DC. Gray histograms represent isotype controls. Graphs shown are representative of four individual experiments.

Specialized dendritic cells (DCs) can endogenously generate Foxp3^+^ iTregs and DC-derived signals have been implicated to contribute to a stable Foxp3 expression [18]. By using both DC-supplemented and APC-free *in vitro* cultures we aimed to define conditions resulting in differentiation of Foxp3^+^ iTregs with maximum suppressive capacity and comparative stability. CD4^+^eGFP^−^ T cells sorted from DEREG mice to a high purity (Figure S1) were used to generate eGFP^+^Foxp3^+^ iTregs that could be easily isolated by FACS sorting on the basis of eGFP expression for their functional analysis. We have recently employed a similar approach to generate and characterize CD8^+^Foxp3^+^ T cells [19]. While the vast majority of *ex vivo* isolated Foxp3^+^ Tregs co-express eGFP in DEREG mice (Figure 1A, left panel) [5], we surprisingly detected a sizeable fraction of eGFP^+^Foxp3^−^ and eGFP^−^Foxp3^+^ populations in iTreg cultures supplemented with transforming growth factor-β (TGF-β, retinoic acid (RA), soluble anti-CD3 antibody and GM-CSF derived BMDC (Figure 1A). Albeit the frequency of eGFP^+^Foxp3^+^ cells peaked by day 3 of the differentiation, we could obtain maximum absolute numbers of eGFP^+^Foxp3^+^ cells by day 4, and further differentiation led to a drastic decline in eGFP^+^Foxp3^+^ cell frequencies (Figure S2).

**Figure 2:**
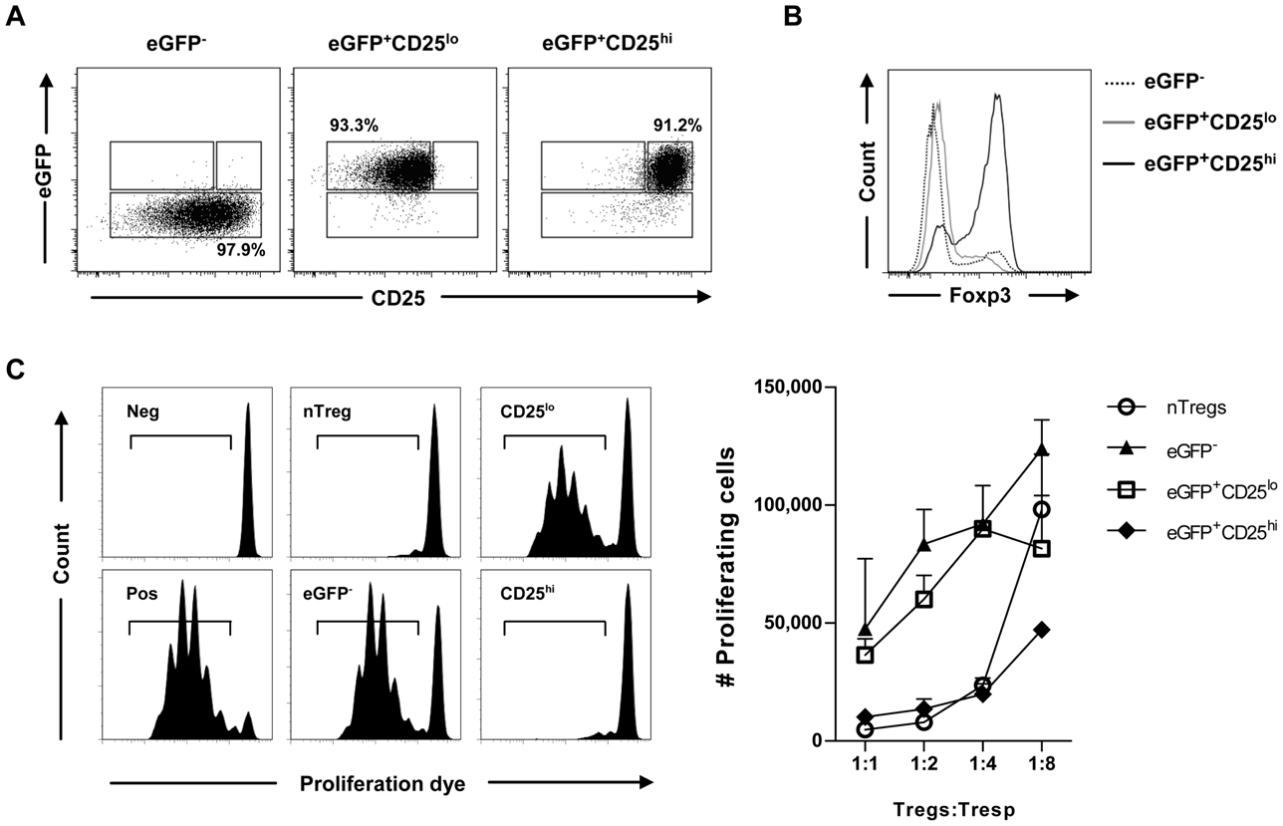
eGFP^+^Foxp3^−^ T cells lack suppressive activity. FACS-sorted CD4^+^eGFP^−^, CD4^+^eGFP^+^CD25^lo^ and CD4^+^eGFP^+^CD25^hi^ populations were added at varying ratios to responder T cells (Tresp) with simultaneous anti-CD3 stimulation. *Ex vivo* isolated CD4^+^CD25^+^eGFP^+^ cells (nTregs) from DEREG mice were used as control. (A) Dot plots demonstrate the purity of various FACS-sorted iTreg populations. Sorting was performed on the basis of eGFP and CD25 expression. (B) Comparison of Foxp3 expression on sorted iTreg sub-populations. Dotted line represents Foxp3 expression on live CD4^+^eGFP^−^ T cells, solid gray line represent Foxp3 expression on live CD4^+^eGFP^+^CD25^lo^ and solid black line represent Foxp3 expression on CD4^+^eGFP^+^CD25^hi^ T cells. (C) Representative histograms for dilution of proliferation dye on gated live CD4^+^ Tresp cells (left panel). Quantification of proliferated Tresp cells under various conditions (right panel). Stimulated and non-stimulated Tresp cells served as positive (Pos) and negative (Neg) controls, respectively. Error bars designate SD of triplicates from one representative of three individual experiments.

As the viable isolation of Foxp3^+^ iTregs relies on eGFP expression we pursued further characterization of eGFP^+^Foxp3^−^ cells which would potentially contaminate the FACS-sorted eGFP^+^ iTreg fraction. To investigate if the eGFP^+^Foxp3^−^ cells are diverted to other helper T cell lineages, we performed intra-cellular staining for Th1, Th2 and Th17 signature cytokines i.e. IFN-γ, IL-13 and IL-17A, respectively. A reasonable fraction of eGFP^+^Foxp3^−^ cells showed the induction of IFN-γ (Figure S3). Interestingly, within the same culture, the eGFP^+^Foxp3^+^ cells did not show production of any of the three cytokines tested, implying commitment to the Treg cell lineage. Next, we assessed for differential expression of various Treg associated surface markers. Amongst the surface antigens tested (PD-1, Nrpl-1, GITR, CD127 and CD25), clearly higher expression of only CD25 correlated with Foxp3 expression within the CD4^+^eGFP^+^ iTreg population (Figure 1B, upper panel). Additionally, the Foxp3^+^ fraction of CD4^+^eGFP^−^ T cells also demonstrated higher CD25 expression (Figure 1B, lower panel). Concomitantly, viable eGFP^+^Foxp3^−^ cells could be enriched based on low CD25 expression by FACS sorting, thereby allowing further functional characterization of this unexpected population (Figure 2A). In a classical *in vitro* T cell suppression assay, CD4^+^eGFP^+^CD25^hi^ cells demonstrated efficient inhibition of T cell proliferation in a dose dependent manner (Figure 2C). This inhibition was comparable to the suppression shown by CD4^+^eGFP^+^CD25^+^ Tregs isolated directly *ex vivo* from secondary lymphoid organs of DEREG mice. Interestingly, CD4^+^eGFP^+^CD25^lo^ cells did not exhibit significant suppressive activity *in vitro* (Figure 2C). Similarly, activated CD4^+^eGFP^−^ cells lacked suppressive activity (Figure 2C) despite of broad CD25 expression (Figure 2A, left panel). Hence, sorting merely on the basis of surface CD25 expression on CD4^+^ cells would result in a substantial contaminating non-iTreg fraction. This emphasizes the valuable aid provided by the transgenic reporter system.

Interestingly, when plate bound anti-CD3 and anti-CD28 was used for *in vitro* differentiation of iTregs, the majority of differentiated eGFP^+^ cells exhibited Foxp3 expression (Figure 1A, right panel). Only a very minor fraction of the differentiated iTregs demonstrated discordant expression of eGFP and Foxp3 under these conditions. Moreover, the use of Flt3-L derived BMDC instead of GM-CSF derived BMDC, resulted in decreased frequencies of eGFP^+^Foxp3^−^ cells which again exhibited comparatively lower surface CD25 expression in comparison to their eGFP^+^Foxp3^+^ counterparts (data not shown). Thus, we here describe conditions for the optimal *in vitro* generation of eGFP^+^Foxp3^+^ iTregs by the use of DEREG CD4^+^eGFP^−^ T cells. Additionally, non-concordant Foxp3 and transgenic eGFP expression could still be surpassed by exploiting the magnitude of CD25 surface expression coupled with BAC-encoded eGFP expression in iTregs. These results are of immense technical importance and of specific relevance for further exploring the basis of differential gene regulation at distinct Foxp3 loci under defined *in vitro* conditions.

## Discussion

During the screening for conditions that result in the generation of stable and highly suppressive CD4^+^Foxp3^+^ iTregs using DEREG mice, we observed unexpected eGFP^+^Foxp3^−^ and eGFP^−^Foxp3^+^ populations. eGFP^+^Foxp3^−^ T cells expressed lower levels of CD25 when compared with their eGFP^+^Foxp3^+^ counterparts, consistent with CD25 being a direct target gene of Foxp3 [20]. We could thus utilize the intensity of CD25 expression to isolate and characterize eGFP^+^Foxp3^−^ T cells. In contrast to eGFP^+^CD25^hi^ iTregs, eGFP^+^CD25^lo^ T cells lacked significant suppressive activity. This is consistent with previous studies demonstrating that, Foxp3 is essential to confer suppressive activity. Given that IL-2 is an important T cell growth factor, increased consumption of IL-2 by eGFP^+^CD25^hi^ cells could be a simple explanation for their higher suppressive activity. Similarly, the eGFP^−^ fraction, predominantly comprising of CD25^lo^ T cells, lacked suppressive activity. However, as CD25 is only one of about 700 Foxp3 target genes that may be involved in conferring suppressive properties [21], additional mechanisms might be involved. The complete lack of suppressive activity by the eGFP^−^ population, albeit containing a small fraction of eGFP^−^Foxp3^+^ T cells could be simply explained by the minor proportion of Foxp3^+^ cells present in this fraction. Alternatively, a non-suppressive behavior of the eGFP^−^Foxp3^+^ population could be hypothesized, as despite the successful Foxp3 induction, these cells may not have fully established the Foxp3-dependent suppressive program. In line with this notion, small populations of non-suppressive Foxp3^+^ T cells have been reported in mice [22].

While eGFP^+^Foxp3^−^ and eGFP^−^Foxp3^+^ populations are not prominently observed in DEREG mice, chronic DT treatment of DEREG mice results in the outgrowth of DT resistant eGFP^−^Foxp3^+^ Tregs [23],[24]. This may be explained by the progressive expansion of few Foxp3^+^ Tregs that have silenced the BAC transgene. However, no selective pressure existed under defined *in vitro* culture conditions, and also, selective silencing of the BAC transgene would contradict the observation of emergence of the eGFP^+^Foxp3^−^ population. Given that the eGFP^−^Foxp3^+^ cells were not prominently induced following plate-bound stimulation with anti-CD3, which induces a similar if not a stronger degree of proliferation, it appears more likely that T cell-intrinsic events induced by the mode and/or magnitude of TCR stimulation along with TGF-β-induced signals *in vitro* are responsible for the differential expression of the BAC-encoded and the endogenous chromosomal Foxp3 loci. Indeed, stimulation by soluble versus plate-bound anti-CD3 has been implicated to induce distinct signaling events downstream of the TCR [25],[26]. T cell activation by soluble anti-CD3 could result in a suboptimal induction of regulatory factors vital for Foxp3 expression, leading to their competitive binding to the distinct Foxp3 loci and resulting in single positive populations in a proportion of developing iTregs. The underlying mechanisms for the observed *in vitro* phenomenon need further elucidation. In addition to the suboptimal TCR/TGF-β signal transduction, the influence of position-effect variegation due to multiple integration of BAC transgene and the accessibility of the different Foxp3 loci may vary, or the stability of Foxp3 and eGFP expression may be differentially regulated in a proportion of cells by post-transcriptional, post-translational or epigenetic mechanisms. Whether these effects represent a direct consequence of the mode of TCR stimulation or involve indirect signals from secreted factors or accessory cells, remains yet to be defined.

In summary, our study illustrates that Foxp3 and eGFP expression which is normally highly concordant in DEREG mice, can be uncoupled during certain conditions of *in vitro* stimulation. This can be circumvented by the use of CD25 as an additional marker for the isolation of highly suppressive CD4^+^Foxp3^+^ iTregs. Additional investigation of this phenomenon may provide further insights into the regulation of Foxp3 gene locus.

## Materials and Methods

### Animals

DEREG mice were bred at the animal facility of Twincore (Hannover, Germany) and at the Helmholtz Centre for Infection Research (HZI, Braunschweig, Germany). 6–12 weeks old sex and age matched mice were used for all experiments. All animals were housed under specific pathogen-free conditions and experiments were performed according to the guidelines approved by institutional, state and federal committees for animal welfare (09-1662). No experiments were conducted on living animals. Mice were sacrificed by cervical dislocation as approved by German animal welfare act and every effort was made to minimize any sort of suffering to the animals.

### Flow Cytometry

The following antibodies were purchased from eBioscience (Frankfurt, Germany): CD4-PE-Cy7 (GK1.5), CD25-PE (PC61.5), PD1-PE (J43), CD127-APC (A7R34), GITR-APC (DTA-1), Streptavadin-PE and functional grade anti-CD3e (17A2) and anti-CD28 (37.51). Biotinylated-Neuropilin-1 polyclonal antibody was purchased from R&D (Wiesbaden-Nordenstadt, Germany). Intracellular staining with Foxp3 (FJK-16s) was performed after fixing and permeabilizing the cells with the Foxp3 Fix/Perm kit (eBioscience) according to the manufacturer's guidelines. Cell proliferation was evaluated using CellTrace™ Violet Cell Proliferation Kit (Life technologies, Darmstadt, Germany). Dead cells were excluded by propidium iodide (Sigma, Munich, Germany) or by ethidium bromide monoazide (Sigma, Munich, Germany) staining prior to the fixation. FACS acquisition was done either on LSR II (Becton Dickinson, Heidelberg, Germany) or CyAn^TM^ ADP (Beckman Coulter, Krefeld, Germany) and data was analysed with FlowJo software (Tree Star, Inc., Oregon, USA).

### In vitro induction of Tregs

Single cell suspensions were obtained from spleens and lymph nodes of DEREG mice by mechanical disruption and passing the cells through 70 µm sieves. RBC lysis was performed with hypotonic lysis buffer containing ammonium chloride. CD4^+^ T cells were enriched with the Dynal® Mouse CD4 Cell Negative Isolation Kit (Invitrogen, Darmstadt, Germany) and further FACS sorted as CD4^+^CD25^−^eGFP^−^ T cells. Sorting was performed at the Cell Sorting Core Facility of the Hannover Medical School on FACSAria (Becton Dickinson, Heidelberg, Germany), XDP or MoFlo (Beckman Coulter, Krefeld, Germany) cell sorters.

For *in vitro* differentiation of iTregs, 2.5×10^4^ CD4^+^CD25^−^eGFP^−^ T cells were co-cultured with 1.0×10^4^ sex matched GM-CSF- or Flt3L-derived bone marrow dendritic cells (BMDC) in a total volume of 200 μl complete RPMI 1640 medium in a 96-well round bottom plate. The cultures were supplemented with 10 nM RA (Sigma-Aldrich, Munich, Germany), 2 ng/mL rhTGF-β1 (Peprotech, Hamburg, Germany), 200 U/mL rhIL-2 (Roche, Germany) and 0.5 µg/mL anti-CD3e (clone-17A2; eBioscience, Frankfurt, Germany). After two days, cells were supplemented with 200 U/mL rhIL-2 and were further incubated for two days at 37°C, ≥95% humidity and 5% CO_2_.

For induction of Tregs in an APC-independent system, 96-well round bottom plates were coated overnight at 4°C with 10 μg/mL anti-CD3e and anti-CD28 antibodies in PBS in a volume of 50 μL per well. The next day plates were thoroughly washed and 2.5×10^4^ FACS sorted CD4^+^CD25^−^eGFP^−^ T cells were seeded per well in 200 μL complete RPMI 1640 medium supplemented with 10 nM RA, 2 ng/mL rhTGF-β1 and 200 U/mL rhIL-2 with further addition of rhIL-2 post two days of culture. Differentiated iTregs were harvested after total of four days culture.

### In vitro proliferation inhibition assay

After four days of cultures iTregs were harvested and surface stained for CD4 and CD25 on ice. Various sub-populations of the iTregs were FACS sorted as CD4^+^eGFP^−^, CD4^+^eGFP^+^CD25^lo^ and CD4^+^eGFP^+^CD25^hi^ cells. CD4^+^eGFP^+^CD25^+^ nTregs and CD4^+^eGFP^−^CD25^−^ responder T cells (Tresp) were obtained *ex vivo* from DEREG mice upon FACS sorting. Tresp cells were labelled with CellTrace™ Violet Cell Proliferation Kit according to the manufacturer's protocol. A total of 5.0×10^4^ Tresp cells were co-cultured with 3.0×10^3^ GM-CSF derived BMDC in a 96 well round bottom plates. nTregs and iTreg subpopulations were added at the ratio of 1∶1, 1∶2, 1∶4 and 1∶8 to the Tresp cells. TCR stimulation was provided by 1 µg/mL soluble anti-CD3e antibody (17A2; eBioscience) for four days. Negative controls lacked anti-CD3e stimulation. On the fourth day of co-culture, proliferation of Tresp cells was evaluated by FACS upon quantification of the cells undergoing proliferation as scored by dilution of CellTrace^TM^ Violet on live CD4^+^ gated cells.

## Supporting Information

Figure S1 **Foxp3 expression in sorted CD4^+^eGFP^−^CD25^−^ cells used to generate iTregs from DEREG mice.** Left panel demonstrates eGFP and Foxp3 expression on live unsorted CD4^+^ enriched T cell population from DEREG mice, and right panel demonstrates the eGFP and Foxp3 expression on live FACS sorted CD4^+^eGFP^−^CD25^−^ T cells which were then used for iTreg differentiation.
(TIF)

Figure S2 **Kinetic analysis of eGFP and Foxp3 expression in in vitro differentiated iTregs with GM-CSF derived BMDC and soluble anti-CD3.** Frequency of live CD4^+^eGFP^+^Foxp3^+^, CD4^+^eGFP^+^Foxp3^−^ and CD4^+^eGFP^−^Foxp3^+^cells were calculated by FACS each day, from initiation of cultures up to day 5. Data presented here is the mean of triplicates analyzed per day from one representative DEREG mouse out of 7 individual mice. Error bars represent the SD of triplicates.
(TIF)

Figure S3 **Phenotyping of differentiated CD4^+^eGFP^+^ cells from iTreg cultures towards various T helper lineages.** iTregs differentiated for 4 days were stimulated with PMA + ionomycin and then stained intra-cellularly for Th1 (IFN-γ), Th2 (IL-13) and Th17 (IL-17A) signature cytokines. FACS plots demonstrate intracellular expression of individual cytokines in live gated CD4^+^eGFP^+^ cells. Data shown here is a representative plot of iTregs from one DEREG mouse from two individual iTreg differentiation cultures.
(TIF)
