# Supplementary figures and images for: Optimal Isolation of Functional Foxp3+ Induced Regulatory T Cells Using DEREG Mice
Source: PLoS One. 2012 Sep 5;7(9):e44760. doi: 10.1371/journal.pone.0044760 (PMC3434173; doi:10.1371/journal.pone.0044760)

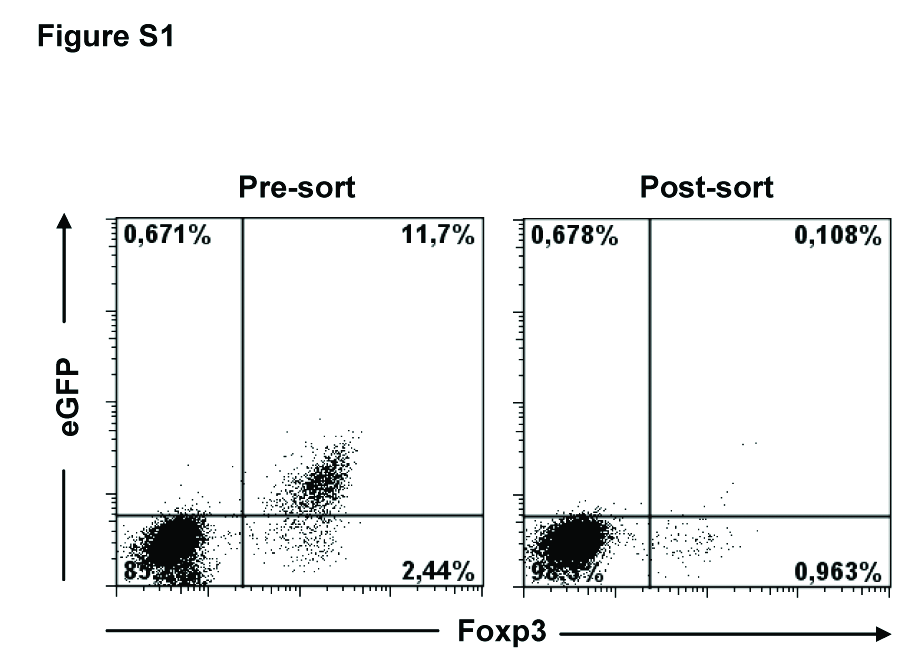

Supplement: Figure S1 — Foxp3 expression in sorted CD4+eGFP−CD25− cells used to generate iTregs from DEREG mice. Left panel demonstrates eGFP and Foxp3 expression on live unsorted CD4+ enriched T cell population from DEREG mice, and right panel demonstrates the eGFP and Foxp3 expression on live FACS sorted CD4+eGFP−CD25− T cells which were then used for iTreg differentiation. (TIF) [file pone.0044760.s001.tif]

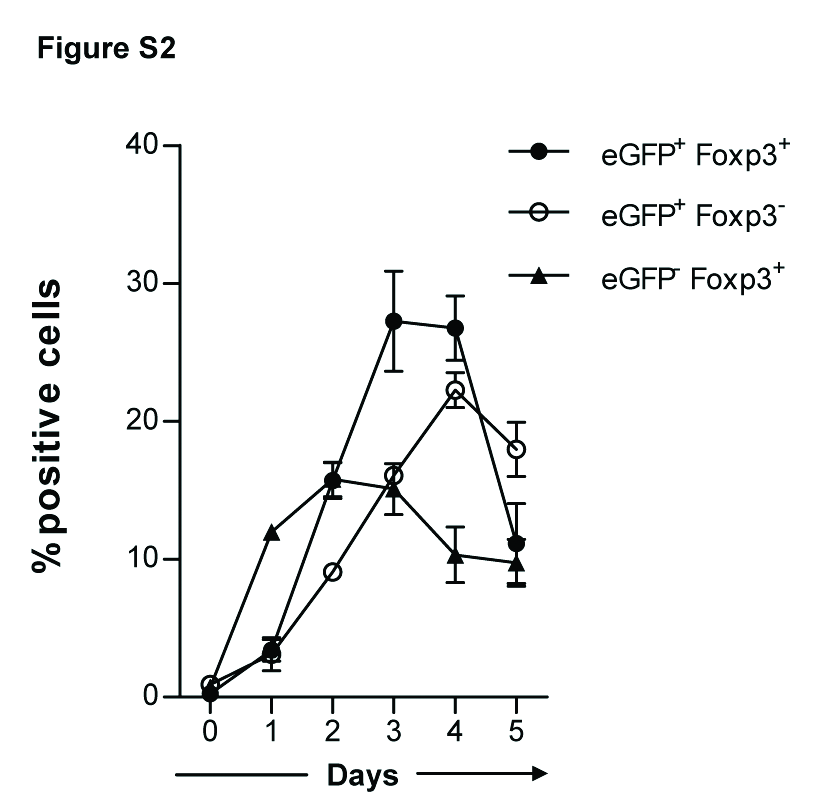

Supplement: Figure S2 — Kinetic analysis of eGFP and Foxp3 expression in in vitro differentiated iTregs with GM-CSF derived BMDC and soluble anti-CD3. Frequency of live CD4+eGFP+Foxp3+, CD4+eGFP+Foxp3− and CD4+eGFP−Foxp3+cells were calculated by FACS each day, from initiation of cultures up to day 5. Data presented here is the mean of triplicates analyzed per day from one representative DEREG mouse out of 7 individual mice. Error bars represent the SD of triplicates. (TIF) [file pone.0044760.s002.tif]

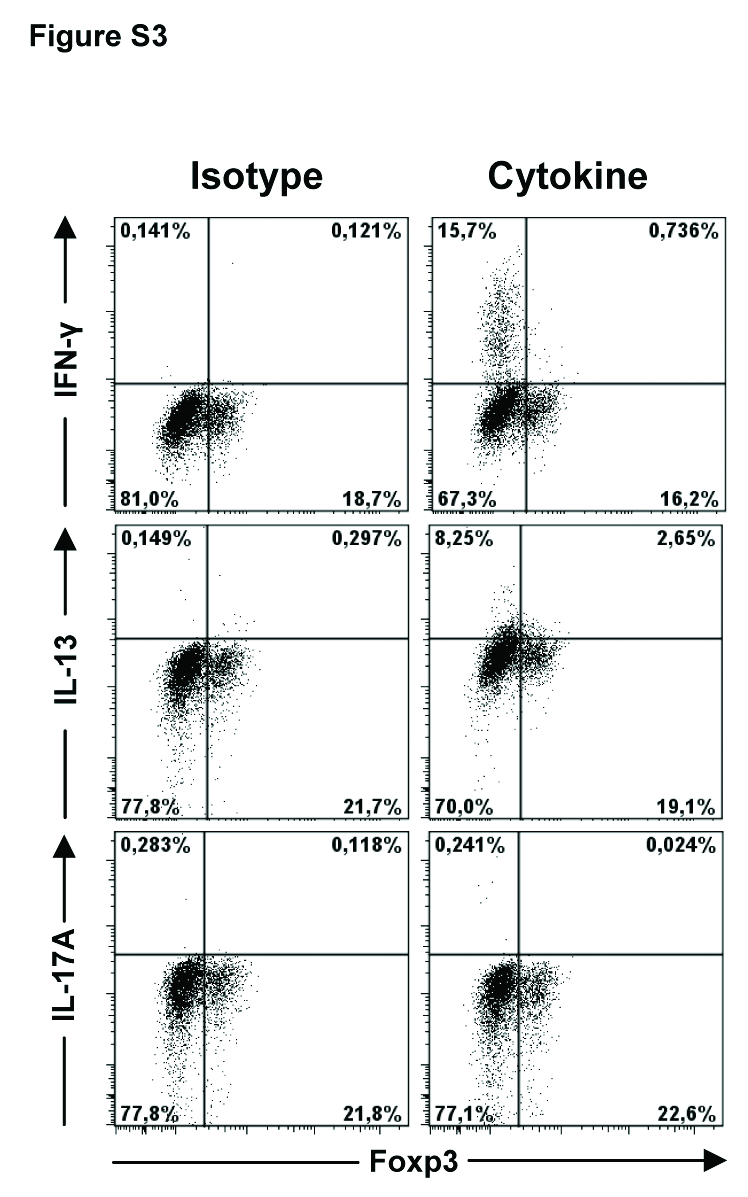

Supplement: Figure S3 — Phenotyping of differentiated CD4+eGFP+ cells from iTreg cultures towards various T helper lineages. iTregs differentiated for 4 days were stimulated with PMA + ionomycin and then stained intra-cellularly for Th1 (IFN-γ), Th2 (IL-13) and Th17 (IL-17A) signature cytokines. FACS plots demonstrate intracellular expression of individual cytokines in live gated CD4+eGFP+ cells. Data shown here is a representative plot of iTregs from one DEREG mouse from two individual iTreg differentiation cultures. (TIF) [file pone.0044760.s003.tif]
